# An Investigation of Flow Patterns and Mixing Characteristics in a Cross-Shaped Micromixer within the Laminar Regime

**DOI:** 10.3390/mi12040462

**Published:** 2021-04-20

**Authors:** Shuai Yuan, Bingyan Jiang, Tao Peng, Qiang Li, Mingyong Zhou

**Affiliations:** 1 College of Mechanical and Electrical Engineering, Central South University, Changsha 410083, China; yuanshuai1006@hotmail.com (S.Y.); jby@csu.edu.cn (B.J.); prettyage@foxmail.com (T.P.); 203711016@csu.edu.cn (Q.L.); 2 State Key Laboratory of High Performance Complex Manufacturing, Central South University, Changsha 410083, China

**Keywords:** computational fluid dynamics, cross-micromixer, numerical diffusion, mixing efficiency, surface roughness

## Abstract

A fast mixing is critical for subsequent practical development of microfluidic devices, which are often used for assays in the detection of reagents and samples. The present work sets up computational fluid dynamics simulations to explore the flow characteristic and mixing mechanism of fluids in cross-shaped mixers within the laminar regime. First, the effects of increasing an operating parameter on local mixing quality along the microchannels are investigated. It is found that sufficient diffusion cannot occur even though the concentration gradient is large at a high Reynolds number. Meanwhile, a method for calculating local mixing efficiency is also characterized. The mixing efficiency varies exponentially with the flow distance. Second, in order to optimize the cross-shaped mixer, the effects of design parameters, namely aspect ratio, mixing angle and blockage, on mixing quality are captured and the visualization of velocity and concentration distribution are demonstrated. The results show that the aspect ratio and the blockage play an important role in accelerating the mixing process. They can improve the mixing efficiency by increasing the mass transfer area and enhancing the chaotic advection, respectively. In contrast, the inflow angle that affects dispersion length is not an effective parameter. Besides, the surface roughness, which makes the disturbance of fluid flow by roughness more obvious, is considered. Three types of rough elements bring benefits for enhancing mixing quality due to the convection induced by the lateral velocity.

## 1. Introduction

In recent years, the lab on a chip (LOC) and micro total analysis system (μTAS) technologies have drawn considerable attention in the detection fields of biology [1], chemistry [2] and medicine [3] due to their unique advantages such as fast analysis speed, less sample consumption, safe operating environment and high throughput [4,5,6]. Compared with conventional macro-scale reactors, the performances of these micro-scale devices are severely restricted by the chemical reaction efficiency, which reduces the assay accuracy. It is known that the mixing of reagents should be done quickly before obvious chemical reaction progress occurs. However, the flow in the microchannel remains laminar and the mass transport process depends mainly on molecular diffusion [7]. Therefore, it is difficult to obtain a uniformly mixed microfluid [8]. Usually, the mixing quality can be improved by increasing the interfacial area for mass transport and by minimizing the diffusion distance [9].

According to the different mixing strategies, micromixers are divided into two categories: active mixers and passive mixers [5]. The former require external energy sources in the form of pressure [10], electric [11,12], acoustic [13], magnetic [14], etc. to create a disturbance inside fluids for enhancing mixing quality. Active mixers generally achieve homogeneous mixing of two or more liquids in a short distance. However, they are less applied in LOC devices because of their drawbacks of high energy consumption and structure complexity [6]. In contrast, the passive mixers only require modification in channel geometries and optimal input parameters to attain better mixing rather than external sources of energy. A high mixing index can be achieved by merely introducing obstacles [15], convergence–divergence patterns [16], curves [17], staggered herringbone structures [18], etc. in the flow paths. For passive mixers, the chaotic convection folds, breaks and stretches the fluids continuously, increasing the mass transfer greatly [19]. To date, passive mixers have been recognized as the most attractive devices. La et al. [20] designed a serpentine passive micromixer that uses the mixing of standard serum and albumin detection reagents to achieve the biochemical detection of albumin levels in samples. Yang et al. [21] reported a passive micromixer with 3D structure. Based on immunofluorescence technology, they employed the antigen–antibody reaction of lung cancer cells to realize the detection and diagnosis of early lung cancer. Lok et al. [22] designed a micromixer with herringbone structures to perform luminol–hydrogen peroxide chemiluminescence detection, and the results showed that the luminescence intensity has a linear relationship with Co^2+^ ion concentration or hydrogen peroxide concentration.

Among passive mixers, T-shaped or cross-shaped microchannels are broadly investigated and are the easiest techniques [23]. Before optimizing these micromixers, it is necessary to understand the flow behavior and mixing mechanism. However, the experimental method is not flexible when the structure of the microchannel needs to be adjusted. Recently, computational fluid dynamics (CFD) has been proven to be a powerful tool for microfluidic design [24]. Detailed looks into local flow fields and transport of various species can be easily visualized with help of CFD analysis. Hence, more and more researchers have focused on flowing and mixing behaviors within micromixers using CFD. Engler et al. [25] reported that the flows in simple T-shaped mixer are characterized by different flow regimes: so-called “stratified (strictly laminar)”, “vortex” and “engulfment” flows. Usually, a good mixing quality can be obtained in the engulfment regime. Soleymani et al. [26] built a three-dimensional (3D) T-shaped mixer to study the influences of operating and design parameters on liquid flow and mixing quality qualitatively. Numerical results showed that the appearance of a vortex was essential to improve mixing performance. Mariotti et al. [27] presented a comparison between experiments and simulations to explore the steady and unsteady regimes in a T-shaped micromixer. The characteristics of the complex 3D vortex structures existing in different regimes were identified in experimental and numerical visualizations. Ault et al. [28] studied the mixing flows in a T-shaped mixer with staggered, offset inlets. They noted that the vortex T-mixer flow exhibited stability characteristics that were tightly coupled to the appearance and evolution of vortex breakdown regions. Wu et al. [29] added vortex-inducing obstacles in the inlet channels of a T-shaped micromixer to improve the mixing quality. In consequence, the performance of the mixer was gradually enhanced with the increase in Reynolds number. Mouheb et al. [30] modeled T-shaped and cross-shaped mixers to explore fluid flow patterns.

Aside from the above research, the influence of surface roughness on microfluid flow cannot be ignored, as the ratio of surface area to volume increases relatively as feature size decreases. Xiao et al. [31,32] studied fluid transport through fibrous porous media with a focus on the effect of surface roughness of capillaries. Dharaiya et al. [33] reported a simulation of flow through straight microchannels with two-dimensional roughness elements. They found that the pressure drops and heat transfer performance in rough channels were enhanced compared with smooth channels. Hu et al. [34] modified rectangular prisms on inner microchannel walls and proved the great effects of roughness element parameters and channel height on velocity distribution and pressure drop.

However, the increasing application of LOC devices requires a deeper understanding of basic transport mechanisms, including fluid flow and mixing behavior inside the microchannels, which is unsatisfactory in the present experimental and numerical studies. The aforementioned simulations were mostly limited to the study of detecting the influences of vortex development and structure optimization in 3D T-shaped or cross-shaped mixers on micromixing within vortex or engulfment regimes and rarely considered the mixing process in a strictly laminar regime. Meanwhile, the effects of surface roughness on mixing have been ignored. Thus, it is necessary to develop numerical methods such as CFD models to solve the above problems.

## 2. Objectives, Model Structure and Numerical Methodology

### 2.1. Objectives

**Figure 1:**
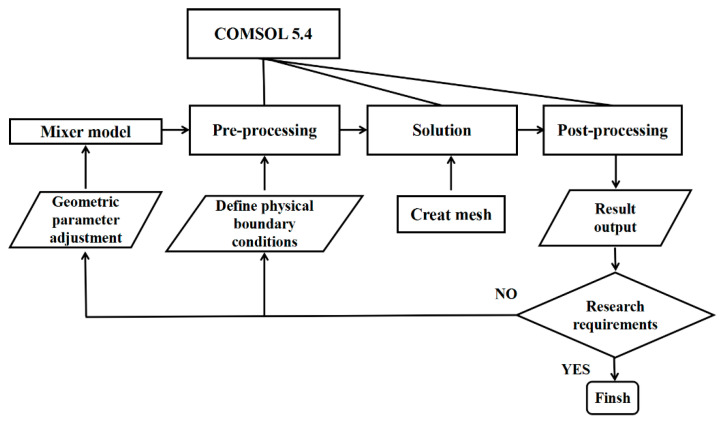
CFD simulation method for a cross-shaped micromixer.

The objective of this work was to explore the mixing mechanism in a cross-shaped mixer for liquid phase application in a stratified regime in which the Reynolds number ranges from 0.1 to 50. Numerical simulations were carried out to investigate the effects of different parameters, including the Reynolds number, aspect ratio, inflow angle and blockage, on flow and mixing performance. Moreover, the effect of surface roughness on mixing was explored as well. There were four main steps in the numerical simulation calculation process, as shown in Figure 1. First, the cross-shaped mixers were built within CFD software or imported from other CAD software. Second, the physical boundary conditions were mainly set at the inlet, outlet and other walls in the preprocessing step. Third, the mesh of the computational domain was generated, and the solution could be calculated in the solution step. Finally, the data of flow field and concentration were extracted from simulation results for further analysis. After completing the simulation of one parameter, the model structure was adjusted and the above process was restarted for the next parameter. This systematic study in microchannels can be extensively used for the design and optimization of micromixers.

### 2.2. Model Structure

**Figure 2:**
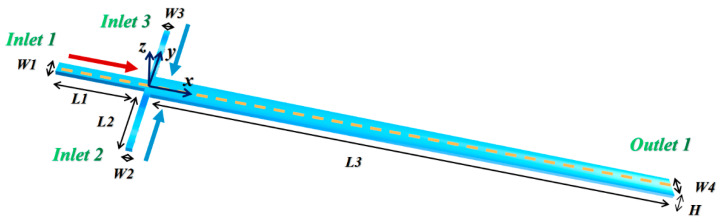
The geometrical structure of the cross-shaped micromixer.

The geometry of the three-dimensional (3D) mixer used in the experiment and numerical simulation in this work is shown in Figure 2, together with the orthogonal rake system. Inlets 2 and 3 have the same rectangular cross-section, with W_1_ = W_3_ = 75 μm. The W_2_ of inlet 1 was set to 150 μm to ensure that different liquids enter mixing channel with equal volume. Their lengths, i.e., L_1_ = L_2_ = 1000 μm, are sufficient to allow a fully developed flow. A W_4_ = 300 μm wide by L_3_ = 10,000 μm long mixing channel exists at the confluence of the three inlet channels. The depth H of the mixer is 60 μm.

### 2.3. Simulation Method and Boundary Conditions

The mixing process of the numerical model was characterized with COMSOL 5.4. For simplifying the flow in microchannels, the compressibility of fluid is usually ignored, and the liquid phase is set to be incompressible. The governing continuity, Navier–Stokes and species convection–diffusion equations, which are used to solve the flow fields and the concentration distributions, are presented as follows:

(1) $$\nabla \cdot \vec{U}=0,$$

(2) $$\rho \left(\vec{U}\cdot \nabla \right)\vec{U}+\nabla P-\mu \nabla^{2}\vec{U}=0,$$

(3) $$\left(\vec{U}\cdot \nabla C\right)=D\nabla^{2}C,$$

where $\vec{U}$, ρ and P stand for the velocity vector, the density of fluid and the pressure, respectively; μ, C and D are the dynamic viscosity, the species concentration and the molecular diffusion coefficient, respectively. The numerical calculations were carried out in two steps: First, Equations (1) and (2) were used to obtain the results of the velocity field, which was substituted into Equation (3). Then, the concentration distribution was solved.

The Reynolds number reflects the ratio of inertial force to viscous force, which is correlated with mixing quality. It can be expressed by the following formula:

(4) $$Re=\frac{\rho UD_{h}}{\mu },$$

where D_h_ is the hydraulic diameter. Here, Re and D_h_ were calculated at the outlet channel. At the microchannel, the viscous effect is dominant due to the small Re. Therefore, the perfect mixing based on turbulence is difficult to achieve in a micromixer. To provide micromixing at the microscale, two mixing mechanisms, namely molecular diffusion and chaotic convection, are applied to passive mixers.

To understand the influence mechanism of different parameters, water and ethanol were injected from three inlets for mixing, with water injected from inlets 2 and 3 and ethanol injected from inlet 1. Their physical properties determined at a constant temperature of 293 K are listed in Table 1 [6]. For boundary conditions, the molar concentration values equaled 1 at inlet 1 and equaled 0 at inlets 2 and 3. A uniform velocity profile was selected as inlet boundary in the computational domain and the zero static pressure was applied at the exit.

### 2.4. Mixing Performance Characterization Method

**Figure 3:**
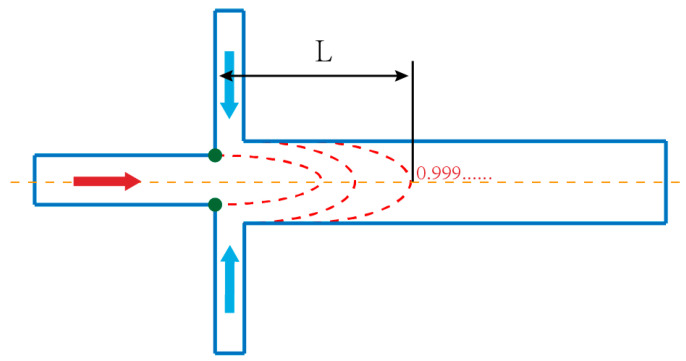
Definition of dispersion length in micromixing.

In order to quantitatively analyze the diffusion rate of species and the extent of mixing at a certain plane, two essential paraments were adopted, namely the dispersion length [35] and the mixing index [7]. As shown in Figure 3, the definition of dispersion length is the distance from the contact point of two liquids to the place where the mass fraction of downstream central sample solution is less than 1. Obviously, a high diffusion rate can result in a short dispersion length; otherwise, it will be relatively long.

The mixing index was adopted to characterize mixing quality at any cross-sections perpendicular to *x*-axis; this index is defined as follows:

(5) $$M.I=1-\frac{\sigma_{m}}{\sigma_{m,\max}},$$

where $\sigma_{m}$ represent the standard deviation of the alcohol molar concentration and $\sigma_{m,\max}$ is the maximum standard deviation of all data ranges. The $\sigma_{m}$ can be calculated as follows:

(6) $$\sigma_{m}=\sqrt{\frac{1}{m}\overset{m}{\underset{j=1}{\sum }}\left(C_{j}-\bar{C}\right)},$$

where $C_{j}$, $\bar{C}$ and m are the mass fraction at selected point j, the mean value of the mass fraction and the number of sample distribution on the transverse plane, respectively. The maximum standard deviation corresponds to a completely unmixed fluid, while the minimum value corresponds to a fully mixed fluid. Hence, the completely mixed fluids are assigned M.I = 1, while completely unmixed fluids are assigned M.I = 0.

## 3. Results and Discussion

### 3.1. Grid Independent and Data Verification

**Figure 4:**
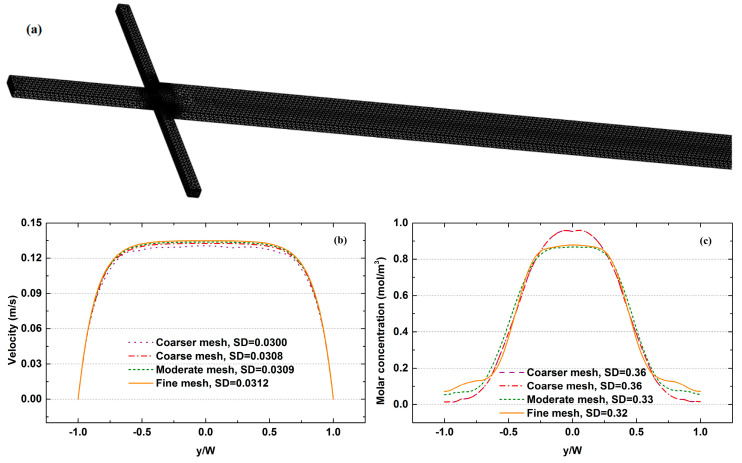
Grid independence verification: (**a**) the tetrahedral grid system used in this work; (**b**) velocity distribution; (**c**) molar concentration distribution.

A mesh refinement analysis was applied to optimize the number of grid nodes, which ensures that the results are independent of the mesh. The unstructured tetrahedral elements shown in Figure 4a were generated with the mesh generator. Four different mesh systems, namely coarser mesh, coarse mesh, normal mesh and fine mesh, were tested with the velocity and concentration calculated at x = 9950 μm, as shown in Figure 4b,c. The standard deviations of four mesh systems (SD) in Figure 4a are 0.0300, 0.0308, 0.0309 and 0.0.0312 mol/m^3^, respectively. When beyond the normal mesh, the influence of increasing the grid number on the accuracy of the results is negligible. A similar conclusion can also be drawn from the results presented in Figure 4c. Therefore, the normal mesh was selected for further calculations.

**Figure 5:**
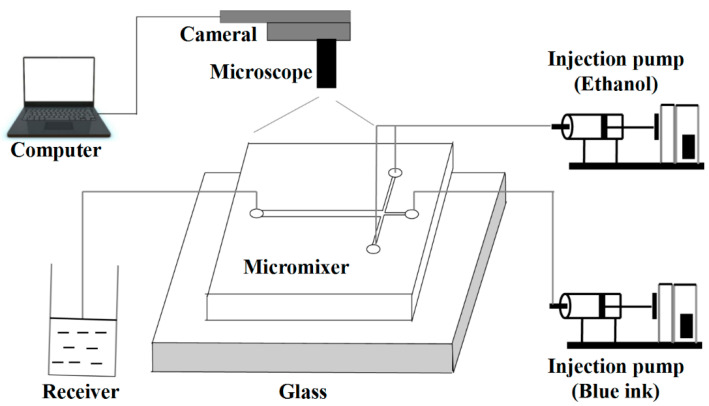
Schematic diagram of the experimental platform.

**Figure 6:**
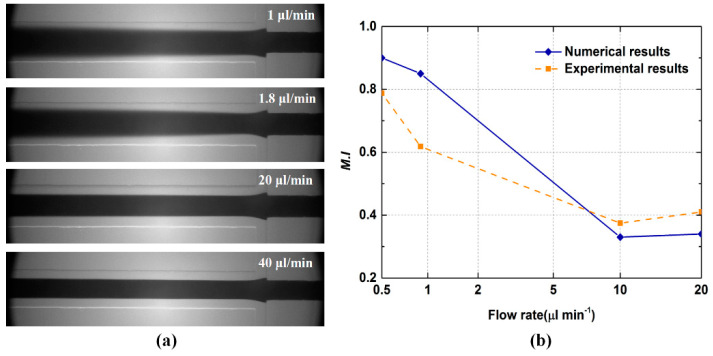
Model validation: (**a**) the grayscale images of the experimental process; (**b**) comparison of experimental data and simulated result of mixing index.

The experimental platform is shown in Figure 5. The micromixer was made of polydimethylsiloxane (PDMS) by the soft lithography method. A closed channel structure was formed by bonding the PDMS micromixer with a glass sheet. Ethanol and blue ink were chosen as the working fluids. The surface roughness of the microchannel was not considered in the experimental process. As seen in Figure 6a, the increase in flow velocity limited the progress of diffusion. Figure 6b presents the comparison between experimental and simulated values. For each case, the mixing indices obtained from simulations were basically in good agreement with the measured mixing indices. It was demonstrated that the above-mentioned numerical models can be used to predict the flow behavior and to understand the mixing mechanism for the following studies.

### 3.2. The Effect of Operating Parameter on Mixing

#### The Effects of Increasing Re on Local Mixing Quality along Microchannel

**Figure 7:**
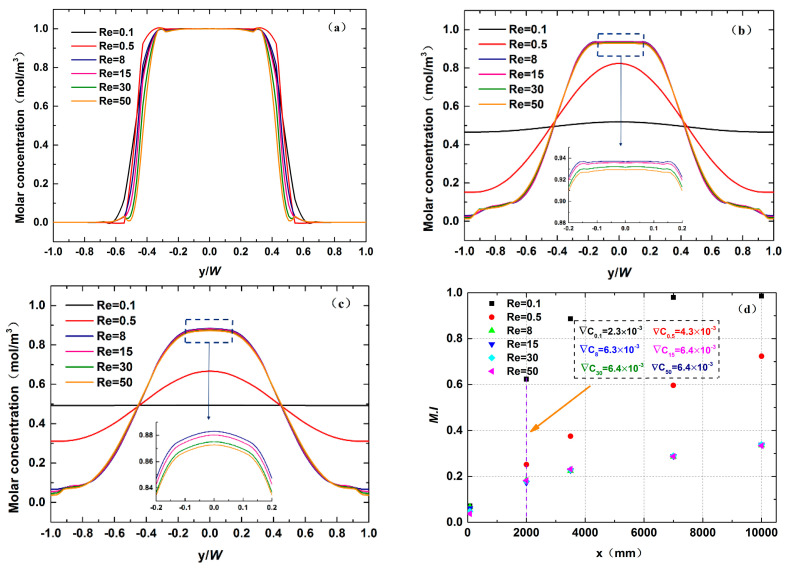
The variations of molar concentration at different cross-sectional positions: (**a**) x = 75 μm, (**b**) x = 5075 μm and (**c**) x = 10,075 μm. (**d**) The performance of mixer at different Re values.

**Figure 8:**
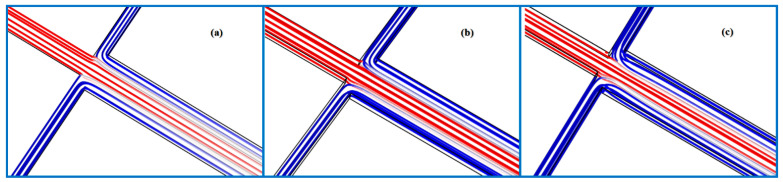
The distributions of streamline at (**a**) Re = 0.1, (**b**) Re = 8 and (**c**) Re = 50.

The developments of species diffusion at three locations along the channel were recorded when the Reynolds number was varied from 0.1 to 50. At x = 75 μm, the liquids have just come into contact, so molecular diffusion has not occurred sufficiently yet. All data curves basically coincide at x = 75 μm, as shown in Figure 7a. As the distance increases continuously, the diffusion under different Re values changes significantly. Figure 7b plots the results at x = 5075 μm. The data profile becomes a straight line with small fluctuations around 0.5 mol·m^−3^ primarily at Re = 0.1. However, it turns into a flat parabola at Re = 0.5, meaning that the mixing quality has been reduced. Beyond Re = 8, there is a little species exchange occurring between the two components. The effects of increasing Re on diffusion seem to be negligible. Similar trends can be observed in Figure 7c. The diffusion phenomenon only develops further after the distance exceeds 7075 μm. Figure 7d represents the variation in the mixing indices as a function of microchannel length. As a whole, by increasing the Re, the mixing quality becomes poorer. At very low Re, i.e., Re = 0.1, the mixing index initially rises rapidly along the *x*-axis direction, and its value reaches 0.9 at a distance of about 4000 μm. Afterward, the mixing efficiency grows slowly. The two components achieve a uniform mixing at the outlet. Figure 8a shows the stable streamline distribution showing that the chaotic advection is not aroused, so the mixing principle at this time depends on molecular diffusion. In another case, namely Re = 0.5, the lower mixing index than that of Re = 0.1 can be associated with the decrease in residence time. An interesting feature in this figure is that the mostly unchanged mixing indices are observed within the same flow path when Re ≥ 8, which is consistent with the development of diffusion discussed in earlier work. The streamlines at these stages do not overlap or become disordered (Figure 8b,c), indicating that the mixing mechanism has not changed. In addition, the 3D vortices near the cross junction induced by the inertial effects do not occur, which is inconsistent with the conclusion of Ault et al. [36]. This can be attributed to the fact that at a very low aspect ratio (0.2), the flow inside the micromixer remains laminar due to the high wall friction of the fluid [26].

Moreover, the local mixing efficiency along the microchannel was characterized. As seen in Figure 7d, the M.I and the distance x maintain a proportional relationship. The least-squares method was used to fit the mixing efficiency of each section:

(7) M.I(x)=1−exp(−κ⋅x),

where κ stands for mixing impact coefficient.

It can be seen that the M.I varies exponentially with the flow distance. The development of mixing is affected by the mixing impact factor κ that is inversely proportional to the flow velocity. The κ can be expressed by Equation (8):

(8) $$\kappa =\frac{\delta }{U},$$

where δ is the mixing coefficient along the microchannel.

Substituting Equation (8) into Equation (7), the formula for the local mixing efficiency along the microchannel can be obtained:

(9) $$M.I\left(x\right)=1-\exp(-\frac{\delta }{U}\cdot x).$$

**Figure 9:**
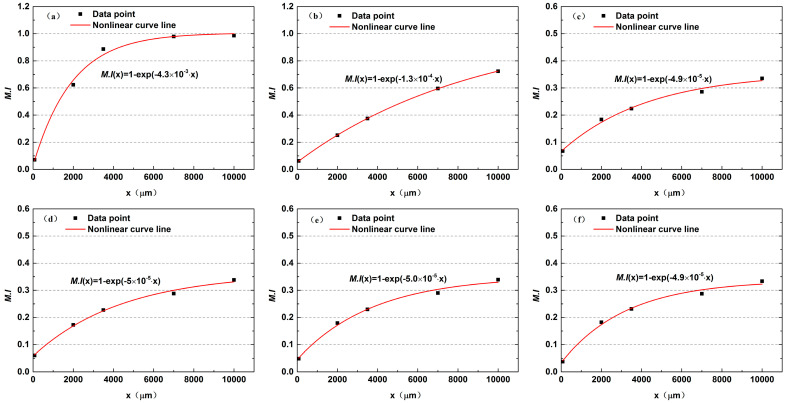
Nonlinear curve fitting of mixing efficiency and distance at (**a**) Re = 0.1, (**b**) Re = 0.5, (**c**) Re = 8, (**d**) Re = 15, (**e**) Re = 30 and (**f**) Re = 50.

Figure 9 shows the fitting results under different Re values. The gradient of all curves gradually decreases along the x-direction. This is because the chemical potential generated by the concentration gradient is the fundamental reason for the movement of molecules from a high-concentration fluid to a low-concentration fluid. In the inlet area, the concentration gradient remains a maximum value, so the mixing efficiency grows quickly. As mixing progresses, the concentration difference becomes smaller, and the driving force of the chemical potential also becomes smaller, resulting in a slower mixing rate in the later stage. However, although the concentration gradient is large at relatively high Re (Figure 7d), poor mixing quality occurs due to the less mixing time. The fluids’ average resident times in the microchannel corresponding to different Re values are listed in Table 2. At the very low Reynold number of Re = 0.1 where it takes 10 s for liquids to flow through the mixer, the two components can achieve uniform mixing, showing that there is a small gap between fluid flow rate and component diffusion rate. When Re varies from 8 to 50, the residence time is not significantly reduced. However, the diffusion rate is much lower than the fluid flow rate. Therefore, the liquids flow out of the channel while the diffusion has not fully developed, which greatly restricts the improvement of the mixing quality. This explains why the fitting curves in Figure 9c–f are similar to each other after Re exceeds 8.

### 3.3. The Effects of Design Parameters on Mixing

#### 3.3.1. The Effect of Aspect Ratio on Mixing

**Figure 10:**
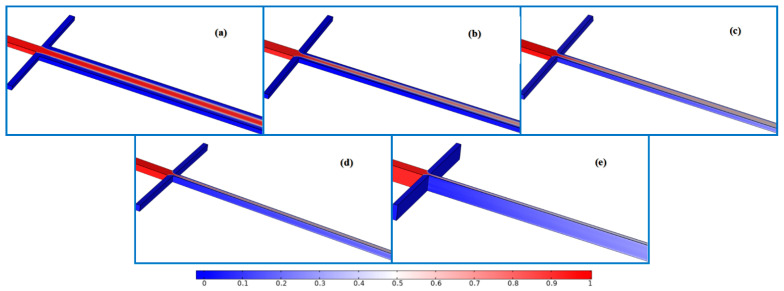
The distributions of concentration under different aspect ratios: (**a**) H/W = 0.2, (**b**) H/W = 0.5, (**c**) H/W = 1, (**d**) H/W = 2, (**e**) H/W = 5.

**Figure 11:**
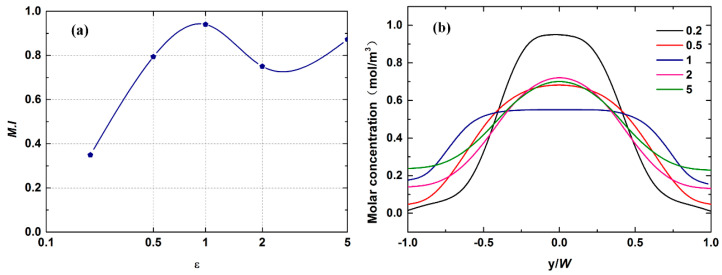
(**a**) The mixing indices under different aspect ratios. (**b**) The molar concentration with different aspect ratios at x = 3000 μm.

This section details the investigation of the effect of microchannel aspect ratio ε, defined as H/W, on mixing efficiency at a fixed Reynolds number of Re = 8. For this purpose, five mixing channel widths, namely 300, 150, 100, 75 and 60 μm, with different ε values (0.2, 0.5, 1, 2 and 5), were constructed. All cases ensured that the hydraulic diameter was 100 μm. The concentration fields under different ε values are shown in Figure 10. As seen from the numerical results, the mixer with ε = 0.2 performs worst in all five cases. The increase in aspect ratio is beneficial for improving mixing quality in the cross-shaped micromixer. Figure 11a displays the mixing indices for each structure. As flagged in this figure, an optimum ε where its corresponding maximum mixing index reaches 0.9 is gained at ε = 1. However, simply increasing ε does not always improve the mixing quality. The mixing efficiency increases first, then decreases and finally increases with the increment in ε. At a low aspect ratio, e.g., ε = 0.2, the mass transfer area between two components is so small that it hinders the full development of diffusion. Thus, the mixing index is very low under the condition of molecular diffusion dominating the mixing process. Before obtaining the optimal aspect ratio (0.2 ≤ ε ≤ 1), the improvement in mixing quality is mainly due to the increment in the contact area and the reduction in diffusion distance along the *y*-axis.

**Figure 12:**
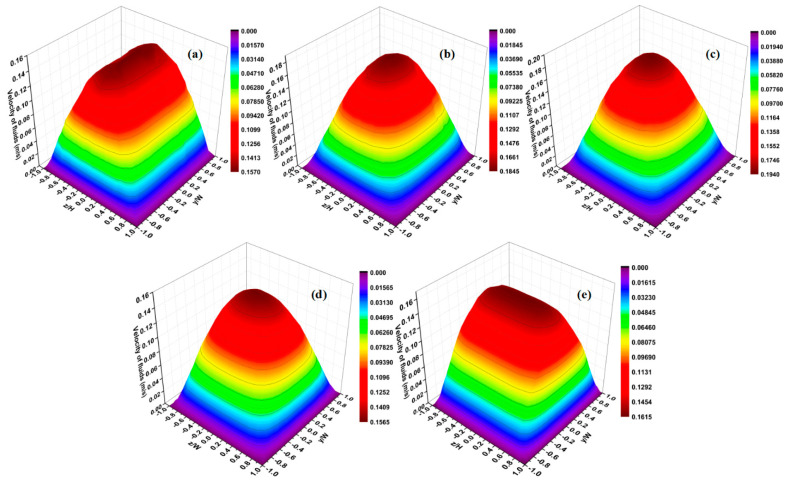
The distributions of velocity under different aspect ratios: (**a**) ε = 0.2; (**b**) ε = 0.5; (**c**) ε = 1; (**d**) ε = 2; (**e**) ε = 5.

According to the theoretical considerations, the achievable gain in the mixing index should still maintain an upward trend after ε = 1. However, the opposite happened. To understand this behavior, the relationship between mass transfer cross-section position and velocity field distribution was assessed. The variations in molar concentration at x = 3000 μm under various ε values are shown in Figure 11b. It can be seen that the maximum and minimum values of curves corresponding to the relatively high aspect ratio have been improved, showing that their performances are much better than that of ε = 0.2, which is consistent with the previous analysis. More importantly, the positions of the contact interface located in the rising and falling stages of curves are considered. Figure 12 finely depicts the velocity field of microfluid in the direction perpendicular to the flow velocity. As the aspect ratio gradually increases from 0.2 to 5, the velocity distribution gradually changes from a flat distribution along the *y*-axis to a flat distribution along the *z*-axis. This makes the mass transfer surface close to the high-speed area at relatively high ε = 2, thereby shortening the resident time in the mixer. However, the contact area at ε = 5 is further increased, so the mixing index begins to improve again, as shown in Figure 11a. Table 3 lists the flow velocity at the initial mass transfer area position. These results confirm the above influence process.

#### 3.3.2. The Effect of Inflow Angle on Mixing

**Figure 13:**
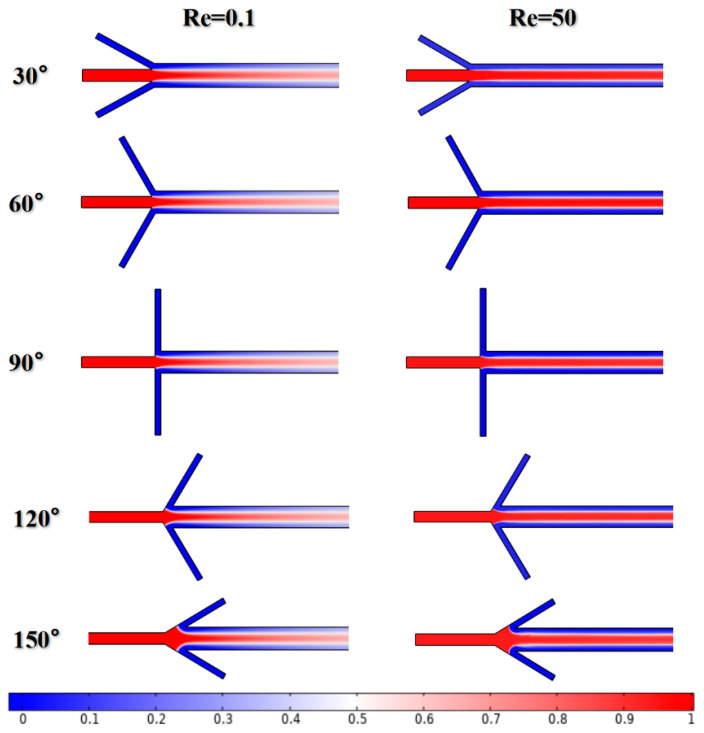
The molar concentration distributions with different inflow angles.

Mixers with inflow angles α ranging from 30 to 150° were built to investigate the effect of α on the mixing performance when Re = 0.1 and 50. All physical parameters except α remain the same as before during the simulation. The concentration fields under different α values are shown in Figure 13. The results show that the effect of inflow angles on mixing quality is weaker than that of Re.

**Figure 14:**
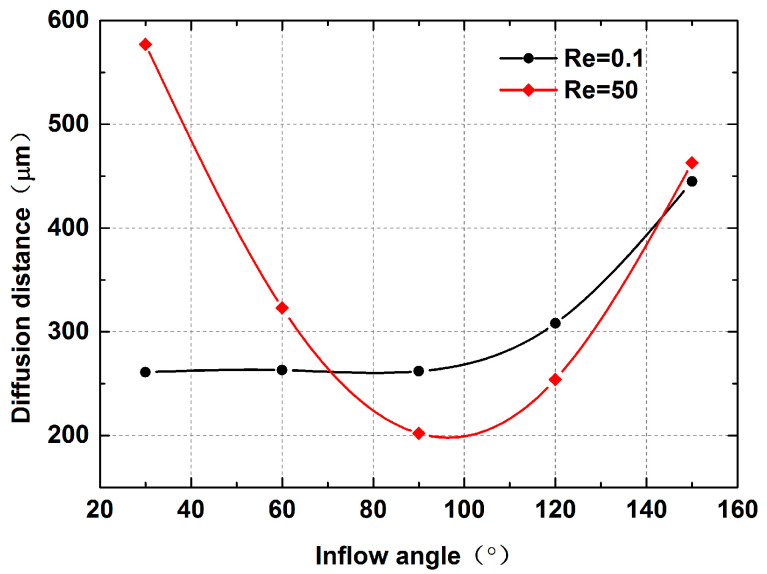
The diffusion lengths with different inflow angles.

**Figure 15:**
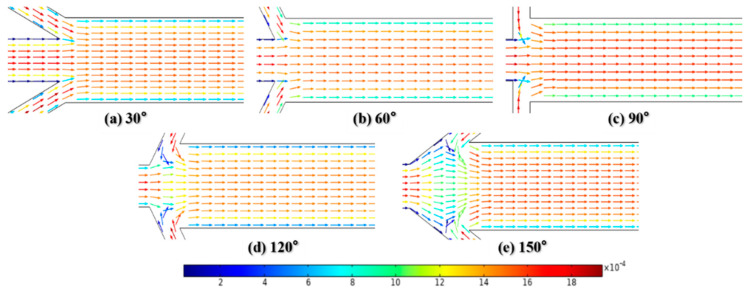
The distribution of velocity vector at Re = 0.1.

**Figure 16:**
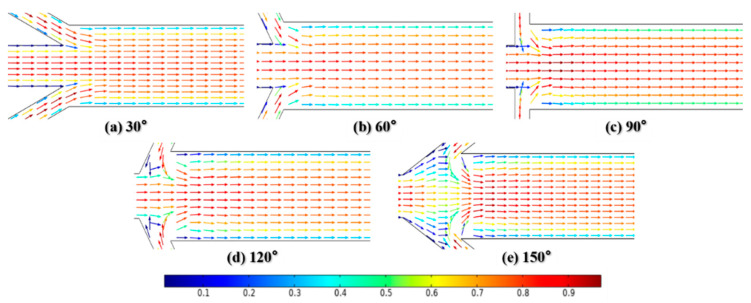
The distribution of velocity vector at Re = 50.

Dispersion length was recorded to reflect diffusion capacity at Re = 0.1 and 50, as shown in Figure 14. At the very low Reynolds number of Re = 0.1, the variation in inflow angles has no significant effect on mixing. The dispersion length is supposed to be about 260 μm when α is less than 90°. This can be attributed to the fact that the flow in the mixing channel remains steady and the convection caused by fluids with low velocity in inlets 2 and 3 is not fully aroused, as shown in Figure 15a–c. Meanwhile, the corresponding results at Re = 50 show a different conclusion. The strong impact caused by the high flow velocity is enhanced with the increase in inflow angle, as seen in Figure 16a–c. Therefore, the dispersion length sharply decreases along the *x*-axis. The *y*-axis values continue to increase regardless of whether the Reynolds number is 0.1 or 50 when α > 90°. Figure 15d,e and Figure 16d,e give the velocity vector distributions of two liquids at Re = 0.1 and 50, respectively. For the large inflow angles, i.e., α = 120 or 150°, where the fluids in the inlets 2 and 3 flow along the *x*-axis in the negative direction, the cross-chip junction area produces greater resistance than other cases. Considering the evidence shown in Figure 13, there may be two reasons leading to the increment in dispersion length. On the one hand, the lateral diffusion distance becomes longer due to the large amount of liquid entering the junction, which cannot be available when α ≤ 90°. On the other hand, the velocity vector sum indicates that the inflow angle is negatively associated with the velocity component along the *y*-axis; therefore, a larger inflow angle induces weaker convection.

Moreover, the effects of inlet angle on the pressure drop and energy consumption were explored. The required pumping power Φ [37] to operate mixers can be calculated as follows:

(10) Φ=Q⋅ΔP,

where Q is the flow rate and ΔP is the pressure drop.

Table 4 lists the results of ΔP and Φ at Re = 0.1 and 50. The increase in the inflow angle does not cause a sharp increase in ΔP, which also means that Φ does not change sharply.

#### 3.3.3. The Effect of Blockage on Mixing

**Figure 17:**
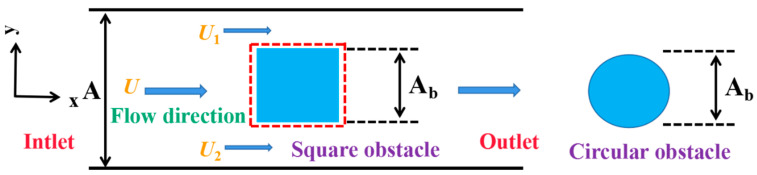
The distribution of obstacles in the microchannel.

Placing obstacles in the microchannel is one of the effective methods for improving mixing quality. The acceleration of fluid flowing through the obstacle in a restricted environment depends on the blockage degree created by the presence of the obstacle [38]. The flow in a confined microchannel cannot be expanded as if it is unconfined. The accelerated flow leads to an augmented Reynolds number in some regions and the generation of chaotic advection, which affects mixing progress. This section describes the results obtained when cylinder and square obstacles, which have often been applied [39,40,41], were used to explore the blocking effect on mixing. Figure 17 shows the distribution of two obstacles in the mixing channel.

The blockage degree is characterized by the ratio of the projected area A_b_ of the obstacle in the width direction of the microchannel to the cross-section area of channel A. Thus, the blockage ratio Ω can be written as follows:

(11) $$\Omega =\frac{A_{b}}{A}.$$

By continuity, the flow velocities U_1_ and U_2_ on both sides of the square obstacle are calculated by the following formula:

(12) $$U_{1}=U_{2}=U\cdot \frac{A}{\left(A-A_{b}\right)}=U\cdot \frac{1}{1-\Omega }.$$

It can be seen that high flow velocity can be achieved by a large blockage ratio.

**Figure 18:**
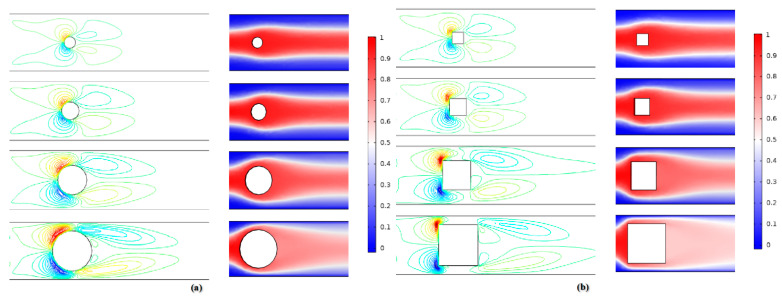
Transverse velocity (left column) and concentration contours (right column) in the micromixers with (**a**) cylinder and (**b**) square obstacles at Re = 50.

**Figure 19:**
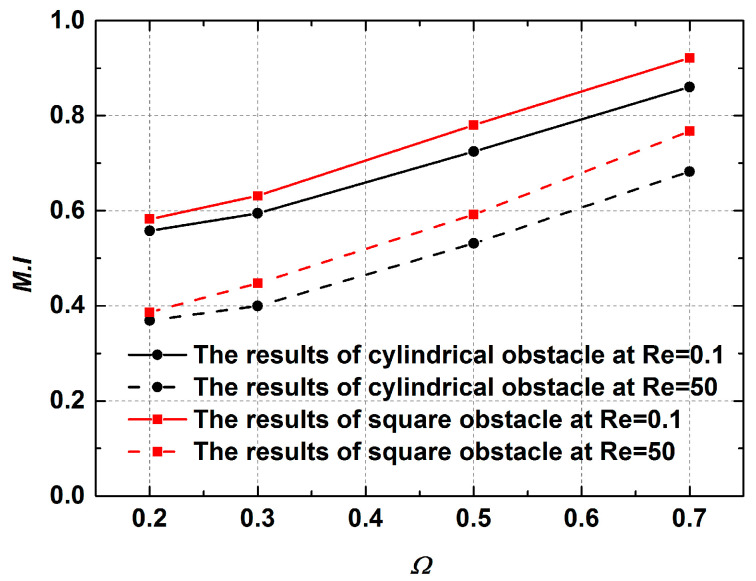
The mixing indices of cylindrical obstacle and square obstacle under different Ω values.

**Figure 20:**
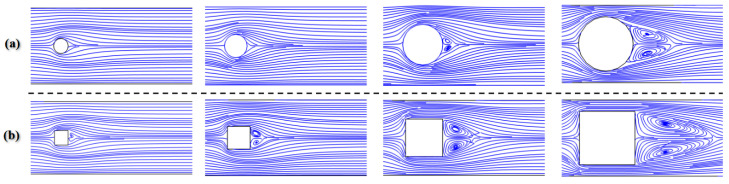
The streamlines of (**a**) cylinder obstacles and (**b**) square obstacles under different Ω values when Re = 50.

Figure 18 shows the contours of transverse velocity and concentration distribution results for different Ω values in the range of 0.2 to 0.7 at Re = 0.1 and 50. As can be seen in this figure, there is a large increase in the distribution of transverse velocity in the mixing channel with the growth of obstruction dimension. This means that the fluids in the middle region move faster toward other fluids, and thus the mixing is enhanced. It can clearly be seen that the performances of mixers with cylinder and square obstacles gradually improve as the Ω increases. The mixing indices at 400 μm downstream of the obstacle were recorded, as shown in Figure 19. At the low Reynolds number of Re = 0.1, the mixing indices of the two cases increase along the x-direction with big gradients. For the performance of the mixer with a square obstacle, the mixing index reaches 0.58 at = 0.2 and exceeds 0.9 at Ω = 0.7, with an increase of 0.32 between them. However, a relatively poor mixing quality is discovered at Re = 50, where the maximum value drops to 0.78 at Ω = 0.7 due to the short mixing time. A similar development tendency can also be seen in the mixer with the cylinder obstacle. Notably, the cylinder obstacle performs worse than the square obstacle both when Re = 0.1 and when Re = 50. This phenomenon can be ascribed to the fact the liquids consisting of two components are continuously squeezed when bypassing the square obstacle. However, the liquids are gradually squeezed when bypassing the circular obstacle. Therefore, the former has a shorter lateral diffusion distance than the latter. In addition, the vortices that form behind the square obstacle also promote the mixing process, as shown in Figure 20. Even though a large blocking ratio improves the mixing quality effectively, it also has an obvious disadvantage in flow resistance, which increases the energy consumption [36].

**Figure 21:**
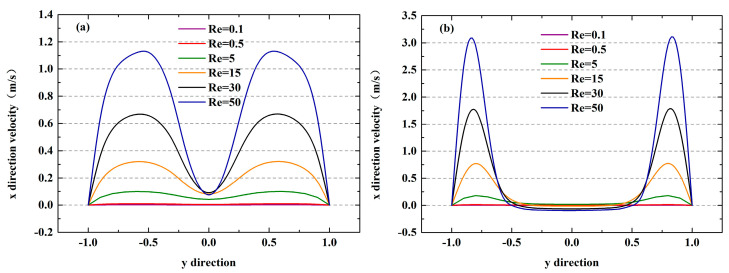
The velocity variation in the x-direction at 50 μm downstream of the obstacle under different Re values: (**a**) Ω = 0.1; (**b**) Ω = 0.7.

The effects of the blockage ratio on the fluid flow state were also characterized. Figure 21 shows the flow rates in the x-direction behind the square obstacle as a function of Re when Ω = 0.1 and 0.7. As illustrated in Figure 21a, the values of velocity are greater than 0 regardless at both high-Re and low-Re conditions, indicating that the vortex caused by the obstacle has difficulty forming when Ω = 0.1. However, a region with a flow rate less than 0 appears when Re > 5, as shown in Figure 21b. This is because a large blockage ratio can form a stronger jet to promote the generation of vortices, which accelerates the flows and causes them to become unsteady.

### 3.4. The Effect of Microchannel Surface Roughness on Mixing

**Figure 22:**
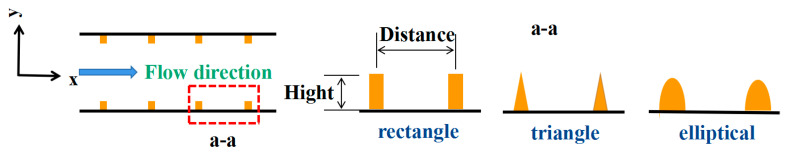
The distribution of standard rough elements in the microchannel.

**Figure 23:**
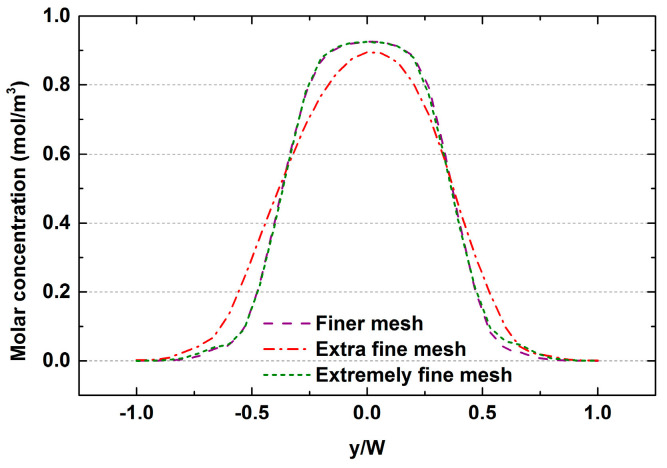
Grid independence verification: molar concentration distribution.

This section describes the investigation of the effect of microchannel surface roughness on mixing. Generally, it is very difficult to simulate the actual roughness structure due to the complexity of the rough wall surface. Generally, standard shape structures are used for approximate simulation in actual research [33,42,43,44]. In this study, 2D models with rectangular, triangular and elliptical rough elements were built, as shown in Figure 22. The distance was kept constant at 100 μm, while the height ranged from 2 to 20 μm. Numerical simulation at a wide Re range (0.1 ≤ Re ≤ 50) was conducted to analyze their effects on mixing. A dimensionless number n, defined as the ratio of the height of the rough element to the width of the microchannel, was used to express the relationship between roughness height and mixing channel width. In order to assess the sensitivity of the solution to the number of computational grids, three mesh systems, namely fine mesh, extra fine mesh and extremely fine mesh, were studied using a mixer with a smooth wall. Figure 23 shows the distribution of mass under different mesh systems at Re = 50. Finally, the extra fine mesh was selected for further calculation.

**Figure 24:**
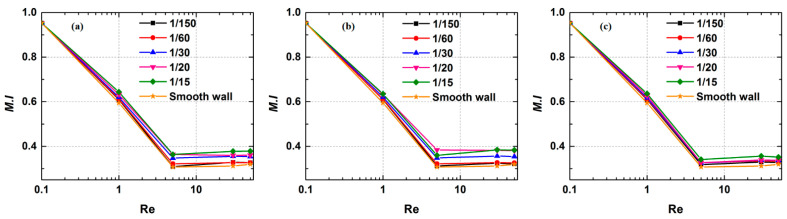
The mixing indices of three mixers with (**a**) square rough element, (**b**) triangular rough element and (**c**) elliptical rough element under different Re values.

**Figure 25:**
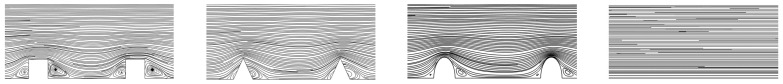
The streamline distributions at Re = 50.

Figure 24 shows the performances of mixers with different rough elements. A similar declining trend in mixing efficiency is seen in each of the three cases. Figure 24a demonstrates the mixing index curves of rectangle roughness element under different Re values at *n* = 1/150, 1/60, 1/30, 1/20 and 1/15. Notably, the differences among the data points at Re = 0.1 are only minor. This finding appears not only in Figure 24b but also in Figure 24c, indicating that both the height and the shape of the roughness element have little effect on mixing at very low Re. However, with the increase in Re, the influence of surface roughness on the mixing is more obvious. The increase in roughness element height is beneficial in improving mixing. For example, the mixing index in a microchannel with a smooth wall is 0.31 at Re = 5, while this value at *n* = 1/15 is increased by 0.056, 0.052 and 0.034 respectively when rectangular, triangular and elliptical rough elements exist. These increases remain basically unchanged with the development of Re. This also shows that the influence of triangular rough elements on the mixing quality is greater than those of rectangular rough elements and elliptical rough elements. Figure 25 shows the velocity streamline diagram in the near-wall area at Re = 50. Vortex areas are formed on both sides of roughness elements, which enhances the impact of the fluids on both sides on the fluids in the middle region. The presence of roughness affects the flow in the area near the wall more when compared with the flow in a smooth microchannel.

## 4. Conclusions

In this study, the performance of a cross-mixer in a strictly laminar regime was assessed to study the flow characteristics and mixing mechanism. A CFD model validated by experimental data was established to explore the influence of Re, aspect ratio, mixing angle, blockage and surface roughness on mixing. The main conclusions of this paper are as follows:

- In the laminar flow regime, molecular diffusion dominates the mixing mechanism. Therefore, the mixing time plays a pivotal role in improving mixing quality. For a low Re, a high mixing index was observed due to the sufficient mixing time. The mixing quality decreased at first and then remained basically unchanged as Re gradually increased to 50. On the whole, the local mixing efficiency along the microchannel showed an exponential development regulation.
- The aspect ratio affects the mixing process by influencing mass transfer area and velocity distribution. The best mixing quality was obtained when the value of the aspect ratio equaled 1. A small aspect ratio caused a poor mixing quality because of the small mass transfer area. For large aspect ratios, although the mass transfer area increased, its position in high velocity region also decreased the mixing efficiency.
- The inlet angle affects the mixing quality by influencing dispersion length. The dispersion length gradually increased when the inflow angle was greater than 90° at Re = 0.1. On the contrary, the dispersion length first decreased and then increased as the inflow angle increased at Re = 50. The optimal inflow angle in a cross-shaped microchannel is about 90°.
- The blocking effect caused by obstacles in a mixing channel enhances the mixing quality. The mixing efficiency was found to increase with the increase in the blocking rate. Moreover, the performance of a mixer with a square obstacle was found to be better than that of a mixer with a cylinder obstacle.
- The presence of surface roughness inside a microchannel promotes the mixing progress. The increase in the height of the roughness element was found to promote the mixing quality. The influence of triangular rough elements on the mixing quality was found to be greater than that of rectangular or elliptical rough elements.

In future work, designing a more efficient planar passive mixer with a short mixing length based on the cross-shaped microchannel is the next stage of research. Meanwhile, some biochemical applications such as heavy metal detection will also be considered.

## Figures and Tables

**Table 1:** Main physical parameters of the two fluids for mixing.

Fluid	Density (kg m^−3^)	Viscosity (kg m^−3^ s^−1^)	Diffusivity (m^2^ s^−1^)
Water	9.998 × 10^2^	0.9 × 10^−3^	1.2 × 10^−9^
Ethanol	7.890 × 10^2^	1.2 × 10^−3^	1.2 × 10^−9^

**Table 2:** The residence time of liquid in mixer under different *Re* values.

Re	0.1	0.5	8	15	30	50
The resident time (s)	10	2	0.125	0.067	0.033	0.02

**Table 3:** The flow velocity at the initial mass transfer area position when ε = 1, 2 and 5.

ε	1	2	5
y/W	0.51	0.39	0.39
V(m/s)	0.099	0.115	0.115

**Table 4:** The pressure drops and the pumping power consumption with different inflow angles.

Re	α (°)	Location	ΔP (kPa)	φ (μW)
0.1	30	Inlet 1	0.039	3.51 × 10^−4^
		Inlets 2 and 3	0.041	1.85 × 10^−4^
	60	Inlet 1	0.040	3.60 × 10^−4^
		Inlets 2 and 3	0.040	1.80 × 10^−4^
	90	Inlet 1	0.040	3.60 × 10^−4^
		Inlets 2 and 3	0.041	1.85 × 10^−4^
	120	Inlet 1	0.038	3.42 × 10^−4^
		Inlets 2 and 3	0.039	1.76 × 10^−4^
	150	Inlet 1	0.040	3.60 × 10^−4^
		Inlets 2 and 3	0.041	1.85 × 10^−4^
50	30	Inlet 1	20.1	90.45
		Inlets 2 and 3	20.3	45.68
	60	Inlet 1	20.0	90.00
		Inlets 2 and 3	20.1	45.23
	90	Inlet 1	20.3	91.35
		Inlets 2 and 3	20.6	46.35
	120	Inlet 1	19.9	89.55
		Inlets 2 and 3	20.0	45.00
	150	Inlet 1	20.3	91.35
		Inlets 2 and 3	20.3	45.68
